# APOE4 Drives Liver/Brain Proteomic Shifts in Young Mice and Alters Metabolic Function Across iPSC‐Derived Hepatic and Neuronal Cells

**DOI:** 10.1002/alz.092482

**Published:** 2025-01-03

**Authors:** Colton R Lysaker, Chelsea N Johnson, Vivien Csikos Drummond, Edziu M Franczak, Cole Birky, Caleb Gilmore, John P Thyfault, Paige C Geiger, Jill K Morris, Heather M Wilkins

**Affiliations:** ^1^ University of Kansas Alzheimer’s Disease Research Center, Fairway, KS USA; ^2^ University of Kansas Medical Center, Kansas City, KS USA

## Abstract

**Background:**

Altered liver function and dysregulated metabolism are emerging risk factors for Alzheimer’s disease (AD). This includes genetic variation in apolipoprotein E (*APOE*), which is the strongest genetic risk determinant for AD. APOE is highly secreted by hepatocytes in the liver and astrocytes in the brain and plays a significant role in lipid homeostasis and metabolic function. Here, we sought to characterize how genetic variation in *APOE* influences hepatic and neuronal metabolic health.

**Method:**

Male and female *APOE3* and *APOE4* targeted replacement mice (Taconic Biosciences) were aged to 4 months of age on a standard chow diet before euthanasia. Liver and brain lysates were analyzed using data‐independent acquisition (DIA) proteomics. Isogenic *APOE3* and *APOE4* human induced pluripotent stem cells (hiPSCs) were differentiated to hepatocytes, astrocytes, and neurons and metabolic function was assessed using Seahorse XF analysis. Additional fluorescence‐based indicators were used to measure reactive oxygen species (ROS), calcium levels, and mitochondrial membrane potential.

**Result:**

APOE4 drove widespread changes to the liver proteome in a sex‐specific manner. Young APOE4 female mice had 402 differentially expressed (DE) proteins when compared to their APOE3 counterparts. These included proteins related to lipid metabolism, mitochondrial dynamics, and mitophagy. Male mice showed far fewer DE proteins in comparison, with APOE4 male mice having 63 DE proteins in comparison to their APOE3 counterparts. Brain proteomics revealed alterations to metabolic pathways including NAD signaling. Additionally, APOE4 iPSC‐derived hepatocytes, astrocytes, and neurons displayed decreased mitochondrial function and increased mitochondrial membrane potential, ROS, and calcium levels.

**Conclusion:**

These results begin to reveal the impacts of *APOE* genetic variation on metabolic function across hepatic and neuronal models. However, underlying mechanistic relationships still need to be elucidated.

